# 5-Fluoro-2-methyl-3-(3-methyl­phenyl­sulfon­yl)-1-benzo­furan

**DOI:** 10.1107/S1600536814006321

**Published:** 2014-03-26

**Authors:** Hong Dae Choi, Pil Ja Seo, Uk Lee

**Affiliations:** a Department of Chemistry, Dongeui University, San 24 Kaya-dong, Busanjin-gu, Busan 614-714, Republic of Korea; b Department of Chemistry, Pukyong National University, 599-1 Daeyeon 3-dong, Nam-gu, Busan 608-737, Republic of Korea

## Abstract

In the title compound, C_16_H_13_FO_3_S, the dihedral angle between the mean planes of the benzo­furan ring system and the 3-methyl­phenyl ring is 80.96 (4)°. In the crystal, mol­ecules are linked *via* pairs of π–π inter­actions between furan and benzene rings, with centroid–centroid distances of 3.758 (1) and 3.771 (1) Å. A similar inter­action is found between furan rings, with a centroid–centroid distance of 3.661 (1) Å between neighbouring mol­ecules. The mol­ecules stack along the *a*-axis direction. In addition, C—H⋯O and C—H⋯π hydrogen bonds are observed between inversion-related dimers.

## Related literature   

For background information and the crystal structures of related compounds, see: Choi *et al.* (2010*a* ▶,*b* ▶, 2012 ▶).
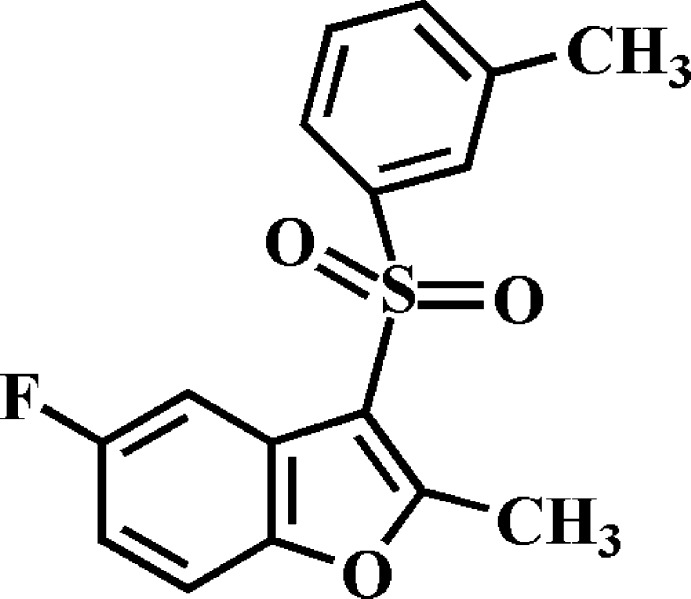


## Experimental   

### 

#### Crystal data  

C_16_H_13_FO_3_S
*M* *_r_* = 304.32
Triclinic, 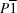

*a* = 7.4406 (1) Å
*b* = 9.1291 (2) Å
*c* = 11.2073 (2) Å
α = 82.891 (1)°
β = 73.301 (1)°
γ = 77.613 (1)°
*V* = 710.62 (2) Å^3^
*Z* = 2
Mo *K*α radiation
μ = 0.25 mm^−1^
*T* = 173 K
0.37 × 0.30 × 0.28 mm

#### Data collection  

Bruker SMART APEXII CCD diffractometer
Absorption correction: multi-scan (*SADABS*; Bruker, 2009 ▶) *T*_min_ = 0.695, *T*_max_ = 0.746
12591 measured reflections
3263 independent reflections
2868 reflections with *I* > 2σ(*I*)
*R*_int_ = 0.026

#### Refinement  

*R*[*F* ^2^ > 2σ(*F* ^2^)] = 0.037
*wR*(*F* ^2^) = 0.103
*S* = 1.07
3263 reflections
192 parameters
H-atom parameters constrained
Δρ_max_ = 0.26 e Å^−3^
Δρ_min_ = −0.41 e Å^−3^

### 

Data collection: *APEX2* (Bruker, 2009 ▶); cell refinement: *SAINT* (Bruker, 2009 ▶); data reduction: *SAINT*; program(s) used to solve structure: *SHELXS97* (Sheldrick, 2008 ▶); program(s) used to refine structure: *SHELXL97* (Sheldrick, 2008 ▶); molecular graphics: *ORTEP-3 for Windows* (Farrugia, 2012 ▶) and *DIAMOND* (Brandenburg, 1998 ▶); software used to prepare material for publication: *SHELXL97*.

## Supplementary Material

Crystal structure: contains datablock(s) I. DOI: 10.1107/S1600536814006321/bg2523sup1.cif

Structure factors: contains datablock(s) I. DOI: 10.1107/S1600536814006321/bg2523Isup2.hkl

Supporting information file. DOI: 10.1107/S1600536814006321/bg2523Isup3.cml

CCDC reference: 993004

Additional supporting information:  crystallographic information; 3D view; checkCIF report

## Figures and Tables

**Table 1:** Hydrogen-bond geometry (Å, °) *Cg*3 is the centroid of the C10–C15 3-methyl­phenyl ring.

*D*—H⋯*A*	*D*—H	H⋯*A*	*D*⋯*A*	*D*—H⋯*A*
C11—H11⋯O2^i^	0.95	2.51	3.450 (2)	172
C6—H6⋯*Cg*3^ii^	0.95	2.76	3.556 (2)	142

Symmetry codes: (i) 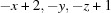; (ii) 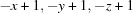.

**Fig. 1.**
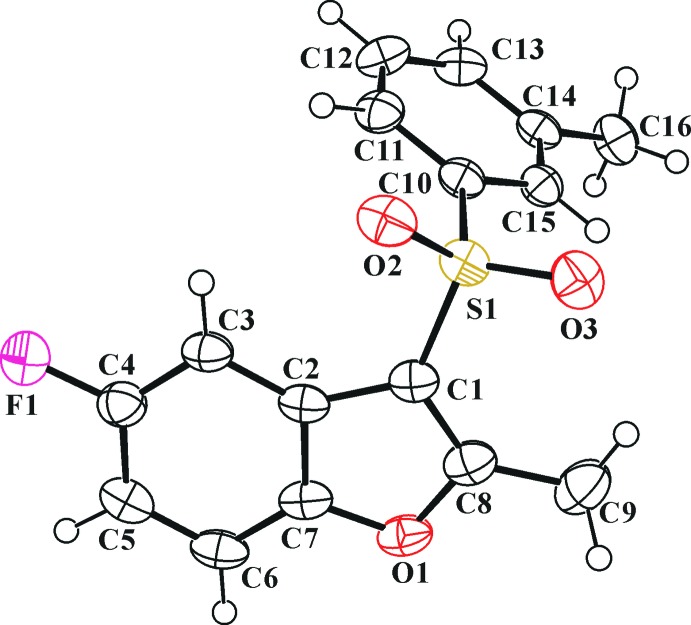
The molecular structure of the title molecule with the atom numbering scheme The displacement ellipsoids are drawn at the 50% probability level. The hydrogen atoms are presented as small spheres of arbitrary radius.

**Fig. 2.**
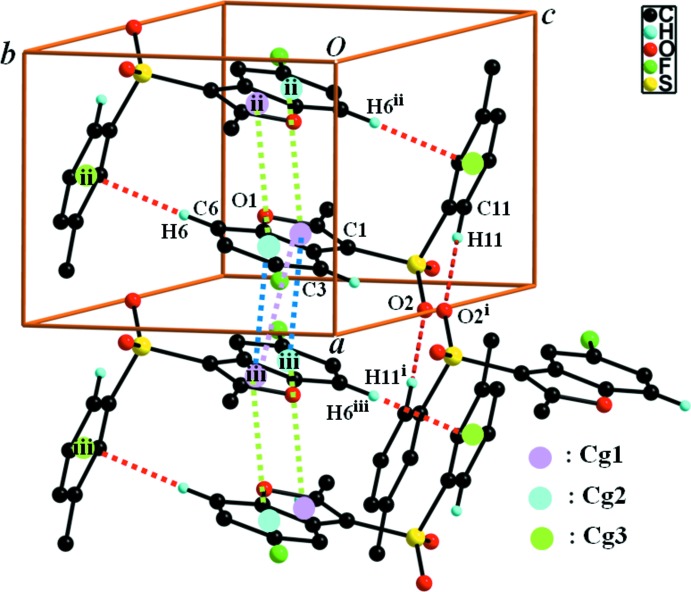
A view of the C—H..O, C—H···π and π···π interactions (dotted lines) in the crystal structure of the title compound. H atoms non-participating in hydrogen-bonding were omitted for clarity. [Symmetry codes: (i) - x + 2, - y, - z + 1 ; (ii) - x + 1,- y + 1,- z + 1; (iii)- x + 2,- y + 1, - z + 1

## supplementary crystallographic information

### 2.1. Synthesis and crystallization

3-Chloro­per­oxy­benzoic acid (77%, 448 mg, 2.0 mmol) was added in small portions to a stirred solution of 5-fluoro-2-methyl-3-(3-methyl­phenyl­sulfanyl)-1-benzo­furan (245 mg, 0.9 mmol) in di­chloro­methane (30 mL) at 273 K. After being stirred at room temperature for 8h, the mixture was washed with a saturated sodium bicarbonate solution and the organic layer was separated, dried over magnesium sulfate, filtered and concentrated at reduced pressure. The residue was purified by column chromatography (hexane–ethyl acetate, 4:1 *v*/*v*) to afford the title compound as a colorless solid [yield 71%, m.p. 375–376 K; *R*_f_ = 0.51 (hexane–ethyl acetate, 2:1 *v*/*v*)]. Single crystals suitable for X-ray diffraction were prepared by slow evaporation of a solution of the title compound in diiso­propyl ether, at room temperature.

### 2.2. Refinement

All H atoms were positioned geometrically and refined using a riding model, with C—H = 0.95 Å for aryl and 0.98 Å for methyl H atoms, respectively. *U*_iso_ (H) = 1.2*U*_eq_ (C) for aryl and 1.5*U*_eq_ (C) for methyl H atoms. The positions of methyl hydrogens were optimized using the SHELXL-97's command AFIX 137 (Sheldrick, 2008).

### 3. Results and discussion

As a part of our ongoing study of 5-fluoro-2-methyl-1-benzo­furan derivatives containing phenyl­sulfonyl (Choi *et al.*, 2010*a*), 4-fluoro­phenyl­sulfonyl (Choi *et al.*, 2010*b*) and 4-methyl­phenyl­sulfonyl (Choi *et al.*, 2012) substituents in the 3-position, we report here on the crystal structure of the title compound.

In the title molecule (Fig. 1), the benzo­furan ring system is essentially planar, with a mean deviation of 0.006 (1) Å from the least-squares plane defined by the nine constituent atoms. The 3-methyl­phenyl ring is essentially planar, with a mean deviation of 0.003 (1) Å from the least-squares plane defined by the six constituent atoms. The dihedral angle formed by the benzo­furan ring system and the 3-methyl­phenyl ring is 80.96 (4)°.

In the crystal structure (Fig. 2), the molecules are linked *via* pairs of π···π inter­actions between the furan and benzene rings, and between the furan rings of neighbouring molecules. The molecules stack along the *a*-axis direction. The relevant centroid names for π···π stacking inter­actions are Cg1 for the furan ring (C1/C2/C7/O1/O8) and Cg2 for the benzene ring (C2–C7). The centroid-centroid separations of Cg1···Cg2^ii^ [(ii): - *x* + 1, - *y* + 1, - *z* + 1], Cg1···Cg2^iii^ and Cg1···Cg1^iii^ [(iii): - *x* + 2,- *y* + 1, - *z* + 1] are 3.758 (1), 3.771 (1) and 3.661 (1) Å, respectively. The slippages of Cg1···Cg2^ii^, Cg1···Cg2^iii^ and Cg1···Cg1^iii^ are 1.227 (1), 1.266 (1) Å and 0.887 (1) Å, respectively. In the crystal packing (Fig. 2), inter­molecular C—H···O and C—H···π hydrogen bonds are observed between inversion-related dimers.

### Crystal data

**Table:**

C_16_H_13_FO_3_S	*Z* = 2
*M**_r_* = 304.32	*F*(000) = 316
Triclinic, *P*1	*D*_x_ = 1.422 Mg m^−^^3^
Hall symbol: -P 1	Melting point = 375–376 K
*a* = 7.4406 (1) Å	Mo *K*α radiation, λ = 0.71073 Å
*b* = 9.1291 (2) Å	Cell parameters from 5506 reflections
*c* = 11.2073 (2) Å	θ = 2.9–27.5°
α = 82.891 (1)°	µ = 0.25 mm^−^^1^
β = 73.301 (1)°	*T* = 173 K
γ = 77.613 (1)°	Block, colourless
*V* = 710.62 (2) Å^3^	0.37 × 0.30 × 0.28 mm

### Data collection

**Table:**

Bruker SMART APEXII CCD diffractometer	3263 independent reflections
Radiation source: rotating anode	2868 reflections with *I* > 2σ(*I*)
Graphite multilayer monochromator	*R*_int_ = 0.026
Detector resolution: 10.0 pixels mm^-1^	θ_max_ = 27.5°, θ_min_ = 1.9°
φ and ω scans	*h* = −9→9
Absorption correction: multi-scan (*SADABS*; Bruker, 2009)	*k* = −11→11
*T*_min_ = 0.695, *T*_max_ = 0.746	*l* = −14→14
12591 measured reflections	

### Refinement

**Table:**

Refinement on *F*^2^	Primary atom site location: structure-invariant direct methods
Least-squares matrix: full	Secondary atom site location: difference Fourier map
*R*[*F*^2^ > 2σ(*F*^2^)] = 0.037	Hydrogen site location: difference Fourier map
*wR*(*F*^2^) = 0.103	H-atom parameters constrained
*S* = 1.07	*w* = 1/[σ^2^(*F*_o_^2^) + (0.056*P*)^2^ + 0.1689*P*] where *P* = (*F*_o_^2^ + 2*F*_c_^2^)/3
3263 reflections	(Δ/σ)_max_ < 0.001
192 parameters	Δρ_max_ = 0.26 e Å^−^^3^
0 restraints	Δρ_min_ = −0.41 e Å^−^^3^

### Special details

**Table:**

Geometry. All esds (except the esd in the dihedral angle between two l.s. planes) are estimated using the full covariance matrix. The cell esds are taken into account individually in the estimation of esds in distances, angles and torsion angles; correlations between esds in cell parameters are only used when they are defined by crystal symmetry. An approximate (isotropic) treatment of cell esds is used for estimating esds involving l.s. planes.
Refinement. Refinement of F^2^ against ALL reflections. The weighted R-factor wR and goodness of fit S are based on F^2^, conventional R-factors R are based on F, with F set to zero for negative F^2^. The threshold expression of F^2^ > 2sigma(F^2^) is used only for calculating R-factors(gt) etc. and is not relevant to the choice of reflections for refinement. R-factors based on F^2^ are statistically about twice as large as those based on F, and R- factors based on ALL data will be even larger.

### Fractional atomic coordinates and isotropic or equivalent isotropic displacement parameters (Å^2^)

**Table:**

	*x*	*y*	*z*	*U*_iso_*/*U*_eq_	
S1	0.87603 (5)	0.18979 (4)	0.70443 (3)	0.03242 (12)	
F1	0.84714 (17)	0.30138 (12)	0.19261 (9)	0.0556 (3)	
O1	0.69505 (15)	0.60973 (11)	0.60126 (11)	0.0391 (3)	
O2	1.02286 (15)	0.10305 (12)	0.61294 (11)	0.0395 (3)	
O3	0.91181 (16)	0.21354 (13)	0.81879 (11)	0.0432 (3)	
C1	0.80671 (19)	0.36430 (15)	0.63233 (14)	0.0310 (3)	
C2	0.79931 (18)	0.39130 (15)	0.50423 (14)	0.0295 (3)	
C3	0.8409 (2)	0.30421 (15)	0.40267 (14)	0.0338 (3)	
H3	0.8885	0.1990	0.4076	0.041*	
C4	0.8087 (2)	0.38018 (17)	0.29498 (15)	0.0382 (3)	
C5	0.7414 (2)	0.53393 (18)	0.28163 (16)	0.0404 (4)	
H5	0.7240	0.5792	0.2037	0.048*	
C6	0.7004 (2)	0.61985 (16)	0.38156 (16)	0.0389 (4)	
H6	0.6545	0.7252	0.3756	0.047*	
C7	0.7292 (2)	0.54522 (15)	0.49103 (15)	0.0333 (3)	
C8	0.7415 (2)	0.49761 (17)	0.68625 (15)	0.0371 (3)	
C9	0.7072 (3)	0.5457 (2)	0.81330 (18)	0.0531 (5)	
H9A	0.5697	0.5784	0.8494	0.080*	
H9B	0.7729	0.6292	0.8091	0.080*	
H9C	0.7562	0.4613	0.8656	0.080*	
C10	0.6704 (2)	0.10864 (15)	0.74169 (13)	0.0300 (3)	
C11	0.6516 (2)	0.01107 (16)	0.66260 (14)	0.0354 (3)	
H11	0.7496	−0.0155	0.5888	0.042*	
C12	0.4846 (2)	−0.04647 (17)	0.69504 (16)	0.0404 (4)	
H12	0.4678	−0.1138	0.6427	0.048*	
C13	0.3430 (2)	−0.00721 (16)	0.80208 (16)	0.0373 (3)	
H13	0.2298	−0.0480	0.8220	0.045*	
C14	0.3615 (2)	0.09077 (15)	0.88184 (14)	0.0319 (3)	
C15	0.5287 (2)	0.14767 (16)	0.85034 (13)	0.0311 (3)	
H15	0.5465	0.2137	0.9034	0.037*	
C16	0.2048 (2)	0.13571 (19)	0.99669 (14)	0.0414 (4)	
H16A	0.1115	0.2202	0.9746	0.062*	
H16B	0.2589	0.1658	1.0579	0.062*	
H16C	0.1415	0.0505	1.0327	0.062*	

### Atomic displacement parameters (Å^2^)

**Table:**

	*U*^11^	*U*^22^	*U*^33^	*U*^12^	*U*^13^	*U*^23^
S1	0.02348 (19)	0.0338 (2)	0.0379 (2)	−0.00384 (14)	−0.00764 (15)	0.00143 (14)
F1	0.0765 (8)	0.0477 (6)	0.0407 (5)	−0.0112 (5)	−0.0125 (5)	−0.0050 (4)
O1	0.0349 (6)	0.0270 (5)	0.0518 (6)	−0.0042 (4)	−0.0050 (5)	−0.0076 (4)
O2	0.0261 (5)	0.0355 (5)	0.0482 (6)	0.0016 (4)	−0.0033 (5)	0.0008 (5)
O3	0.0350 (6)	0.0538 (7)	0.0446 (6)	−0.0116 (5)	−0.0163 (5)	0.0018 (5)
C1	0.0224 (6)	0.0298 (7)	0.0390 (7)	−0.0061 (5)	−0.0045 (6)	−0.0022 (5)
C2	0.0202 (6)	0.0248 (6)	0.0407 (7)	−0.0046 (5)	−0.0047 (6)	0.0014 (5)
C3	0.0300 (7)	0.0251 (6)	0.0421 (8)	−0.0036 (5)	−0.0050 (6)	−0.0009 (6)
C4	0.0370 (8)	0.0356 (7)	0.0403 (8)	−0.0089 (6)	−0.0062 (7)	−0.0025 (6)
C5	0.0354 (8)	0.0374 (8)	0.0460 (9)	−0.0085 (6)	−0.0112 (7)	0.0091 (7)
C6	0.0309 (8)	0.0252 (7)	0.0568 (10)	−0.0034 (6)	−0.0106 (7)	0.0058 (6)
C7	0.0230 (7)	0.0253 (6)	0.0478 (8)	−0.0043 (5)	−0.0034 (6)	−0.0036 (6)
C8	0.0279 (7)	0.0357 (7)	0.0464 (8)	−0.0093 (6)	−0.0042 (6)	−0.0065 (6)
C9	0.0540 (11)	0.0512 (10)	0.0535 (10)	−0.0120 (8)	−0.0058 (9)	−0.0193 (8)
C10	0.0257 (7)	0.0277 (6)	0.0346 (7)	−0.0026 (5)	−0.0089 (6)	0.0033 (5)
C11	0.0317 (7)	0.0290 (7)	0.0405 (8)	0.0006 (6)	−0.0056 (6)	−0.0045 (6)
C12	0.0374 (8)	0.0281 (7)	0.0556 (10)	−0.0032 (6)	−0.0113 (7)	−0.0110 (6)
C13	0.0303 (7)	0.0280 (7)	0.0519 (9)	−0.0065 (6)	−0.0093 (7)	0.0009 (6)
C14	0.0282 (7)	0.0289 (7)	0.0354 (7)	−0.0022 (5)	−0.0090 (6)	0.0059 (5)
C15	0.0295 (7)	0.0311 (7)	0.0319 (7)	−0.0031 (5)	−0.0103 (6)	0.0008 (5)
C16	0.0314 (8)	0.0525 (9)	0.0358 (8)	−0.0078 (7)	−0.0045 (6)	0.0025 (7)

### Geometric parameters (Å, º)

**Table:**

S1—O3	1.4319 (12)	C8—C9	1.478 (2)
S1—O2	1.4346 (11)	C9—H9A	0.9800
S1—C1	1.7394 (14)	C9—H9B	0.9800
S1—C10	1.7626 (14)	C9—H9C	0.9800
F1—C4	1.3575 (18)	C10—C11	1.385 (2)
O1—C8	1.368 (2)	C10—C15	1.389 (2)
O1—C7	1.3686 (18)	C11—C12	1.387 (2)
C1—C8	1.357 (2)	C11—H11	0.9500
C1—C2	1.441 (2)	C12—C13	1.377 (2)
C2—C3	1.393 (2)	C12—H12	0.9500
C2—C7	1.3954 (19)	C13—C14	1.393 (2)
C3—C4	1.372 (2)	C13—H13	0.9500
C3—H3	0.9500	C14—C15	1.386 (2)
C4—C5	1.390 (2)	C14—C16	1.499 (2)
C5—C6	1.371 (2)	C15—H15	0.9500
C5—H5	0.9500	C16—H16A	0.9800
C6—C7	1.377 (2)	C16—H16B	0.9800
C6—H6	0.9500	C16—H16C	0.9800
			
O3—S1—O2	119.73 (7)	C8—C9—H9A	109.5
O3—S1—C1	108.28 (7)	C8—C9—H9B	109.5
O2—S1—C1	107.74 (7)	H9A—C9—H9B	109.5
O3—S1—C10	108.10 (7)	C8—C9—H9C	109.5
O2—S1—C10	108.25 (7)	H9A—C9—H9C	109.5
C1—S1—C10	103.57 (6)	H9B—C9—H9C	109.5
C8—O1—C7	107.14 (11)	C11—C10—C15	121.75 (13)
C8—C1—C2	107.50 (13)	C11—C10—S1	120.15 (11)
C8—C1—S1	127.01 (12)	C15—C10—S1	118.09 (11)
C2—C1—S1	125.42 (11)	C10—C11—C12	117.64 (14)
C3—C2—C7	119.40 (14)	C10—C11—H11	121.2
C3—C2—C1	136.00 (13)	C12—C11—H11	121.2
C7—C2—C1	104.60 (13)	C13—C12—C11	120.88 (14)
C4—C3—C2	115.65 (13)	C13—C12—H12	119.6
C4—C3—H3	122.2	C11—C12—H12	119.6
C2—C3—H3	122.2	C12—C13—C14	121.61 (14)
F1—C4—C3	118.38 (14)	C12—C13—H13	119.2
F1—C4—C5	116.81 (15)	C14—C13—H13	119.2
C3—C4—C5	124.80 (15)	C15—C14—C13	117.73 (14)
C6—C5—C4	119.62 (15)	C15—C14—C16	120.97 (13)
C6—C5—H5	120.2	C13—C14—C16	121.30 (14)
C4—C5—H5	120.2	C14—C15—C10	120.38 (13)
C5—C6—C7	116.43 (13)	C14—C15—H15	119.8
C5—C6—H6	121.8	C10—C15—H15	119.8
C7—C6—H6	121.8	C14—C16—H16A	109.5
O1—C7—C6	125.55 (13)	C14—C16—H16B	109.5
O1—C7—C2	110.36 (13)	H16A—C16—H16B	109.5
C6—C7—C2	124.09 (14)	C14—C16—H16C	109.5
C1—C8—O1	110.40 (14)	H16A—C16—H16C	109.5
C1—C8—C9	134.70 (16)	H16B—C16—H16C	109.5
O1—C8—C9	114.88 (14)		
			
O3—S1—C1—C8	−18.19 (15)	C1—C2—C7—C6	179.58 (13)
O2—S1—C1—C8	−149.03 (13)	C2—C1—C8—O1	−0.94 (16)
C10—S1—C1—C8	96.42 (14)	S1—C1—C8—O1	−178.00 (10)
O3—S1—C1—C2	165.25 (11)	C2—C1—C8—C9	177.59 (17)
O2—S1—C1—C2	34.41 (13)	S1—C1—C8—C9	0.5 (3)
C10—S1—C1—C2	−80.14 (13)	C7—O1—C8—C1	0.89 (16)
C8—C1—C2—C3	−178.42 (16)	C7—O1—C8—C9	−177.96 (13)
S1—C1—C2—C3	−1.3 (2)	O3—S1—C10—C11	−149.08 (12)
C8—C1—C2—C7	0.61 (15)	O2—S1—C10—C11	−18.00 (14)
S1—C1—C2—C7	177.72 (10)	C1—S1—C10—C11	96.18 (13)
C7—C2—C3—C4	0.2 (2)	O3—S1—C10—C15	32.04 (13)
C1—C2—C3—C4	179.12 (15)	O2—S1—C10—C15	163.13 (11)
C2—C3—C4—F1	179.80 (13)	C1—S1—C10—C15	−82.69 (12)
C2—C3—C4—C5	0.8 (2)	C15—C10—C11—C12	0.4 (2)
F1—C4—C5—C6	−179.85 (14)	S1—C10—C11—C12	−178.41 (11)
C3—C4—C5—C6	−0.8 (3)	C10—C11—C12—C13	0.1 (2)
C4—C5—C6—C7	−0.2 (2)	C11—C12—C13—C14	−0.2 (2)
C8—O1—C7—C6	179.87 (14)	C12—C13—C14—C15	−0.3 (2)
C8—O1—C7—C2	−0.49 (15)	C12—C13—C14—C16	178.60 (14)
C5—C6—C7—O1	−179.25 (14)	C13—C14—C15—C10	0.8 (2)
C5—C6—C7—C2	1.2 (2)	C16—C14—C15—C10	−178.06 (13)
C3—C2—C7—O1	179.16 (12)	C11—C10—C15—C14	−0.9 (2)
C1—C2—C7—O1	−0.07 (15)	S1—C10—C15—C14	177.93 (10)
C3—C2—C7—C6	−1.2 (2)		

### Hydrogen-bond geometry (Å, º)

Cg3 is the centroid of the C10–C15 3-methylphenyl ring.

**Table:**

*D*—H···*A*	*D*—H	H···*A*	*D*···*A*	*D*—H···*A*
C11—H11···O2^i^	0.95	2.51	3.450 (2)	172
C6—H6···*Cg*3^ii^	0.95	2.76	3.556 (2)	142

Symmetry codes: (i) −*x*+2, −*y*, −*z*+1; (ii) −*x*+1, −*y*+1, −*z*+1.
